# Biomarkers and sepsis severity as predictors of mechanical ventilation and mortality in COVID-19

**DOI:** 10.1016/j.heliyon.2024.e28521

**Published:** 2024-03-25

**Authors:** O. Jiménez-Zarazúa, L.N. Vélez-Ramírez, J.D. Mondragón

**Affiliations:** a Hospital General Zona 21 IMSS, León, Department of Internal Medicine, Mexico; b Escuela Nacional de Estudios Superiores, Unidad León, Universidad Nacional Autonóma de México (UNAM), Leon, Guanajuato, Mexico; c Hospital General León, Department of Radiology, Mexico; d Department of Medicine and Nutrition, Universidad de Guanajuato, Leon, Guanajuato, Mexico; e University of Groningen, University Medical Center Groningen, Department of Neurology, the Netherlands; f Universidad Nacional Autónoma de México, Instituto de Neurobiología, Departamento de Neurobiología Conductual y Cognitiva, Laboratorio de Psicofisiología, Querétaro, Mexico; g San Diego State University, Department of Psychology, Life-Span Human Senses Lab, San Diego, CA, USA

**Keywords:** COVID-19, Hypoalbuminemia, Lactate/albumin ratio, Sepsis, Septic shock, Survival rate

## Abstract

**Introduction:**

Patients with septic shock face an elevated risk of mortality compared to those with sepsis. Several biomarkers, including lactate dehydrogenase, albumin, and lactate/albumin (L/A), have been associated with increased mortality in COVID-19 patients. This study aims to assess the relationship between sepsis, septic shock, and mortality, as well as the need for mechanical ventilation in COVID-19 patients. Demographic, sepsis severity factors, and biomarkers are examined.

**Methods:**

A retrospective case series from June 2020 to March 2021 included 490 patients diagnosed with sepsis or septic shock secondary to SARS-CoV-2 pneumonia. Time-to-event analyses were conducted for mechanical ventilation and mortality. Statistical significance was set at p ≤ .0038. Serum lactate, albumin, lactate/albumin ratio, C-reactive protein, platelet levels, and three sepsis severity scales, (CCI, SOFA, APACHE IV) were assessed.

**Results:**

Sepsis was identified in 352 patients (71.8%), while 138 had septic shock. Patients with septic shock were more likely to require invasive ventilator support. Factors associated with a higher risk of intubation included higher APACHE IV scores, elevated serum albumin levels, and increased L/A ratio. L/A ratio and serum lactate levels demonstrated the best diagnostic accuracy for mechanical ventilation (AUC, 0.964 and 0.946, respectively), mortality (AUC, 0.926 and 0.887, respectively).

**Discussion:**

Increased C-reactive protein, combined with increased serum lactate and a high lactate/albumin ratio, may assist clinicians in identifying COVID-19 patients at risk of mechanical ventilation and mortality upon admission. Optimal cut-off values for lactate (1.45–1.65 mmol/L) and L/A ratio (0.413) can aid in prioritizing medical care for at risk COVID-19 patients.

## 1 Background

Sepsis and septic shock are two worldwide public health entities that have high mortality rates [1]. Sepsis is defined as a life-threatening organ dysfunction caused by a dysregulated host response to an infection, where organ dysfunction is characterized by a greater than two-point increase in the Sequential Organ Failure Assessment (SOFA) score [2]. In septic shock, there is an abnormal distribution of blood flow associated with vasodilation. Vasoplagia results from the release of various innate immune response mediators associated with the infectious process. The aftermath of this immune response involves microvascular and tissue damage that causes oxygen deficiency at a tissular level and abnormal cellular metabolism [3]. Septic shock in adults can be defined as hypotension requiring vasopressor therapy to maintain mean blood pressure (BP) of 65 mmHg or greater while having a serum lactate level greater than 2 mmol/L after adequate fluid resuscitation [2,4].

Among COVID-19 patients, 1.1–30.6% developed septic shock [5,6]. Patients with septic shock exhibit a higher mortality rate (97.6%) than those without septic shock (3.8%). Notably, risk factors associated with a higher mortality rate included disease severity (HR = 15, p < .001), age >65 years (HR = 2.6, p = .012), temperature >39.1 °C (HR = 2.9, p = .047), leukocytosis (HR = 6.9, p < .001), neutrophil count >75 × 10⁹ (HR = 2.4, p = .022), creatine kinase >5 U/L (HR = 1.8, p = .042), glucose >6.1 mmol/L (HR = 7, p < .001), and lactate >2 mmol/L (HR = 22, p < .001) [7]. Furthermore, high concentrations of lactate dehydrogenase and low concentrations of albumin in serum are linked to higher mortality in COVID-19 patients [8].

Recently, two biomarkers have gained momentum as prognostic factors for sepsis and septic shock: lactate dehydrogenase (serum lactate), and lactate/albumin (L/A) ratio. Hyperlactatemia may result from tissue hypoxia or increased glycolysis secondary to an adaptive response to a septic process [9]. Sepsis-associated hyperlactatemia (SAHL) is a strong independent predictor of mortality; however, is no longer viewed as a proxy for tissue hypoxia or anaerobic glycolysis and is involved in mechanisms to facilitate bioenergetic efficiency via lactate oxidation (adrenergic stimulation or activation of the stress response) [10]. Meanwhile, the L/A ratio has recently served as an independent predictor of mortality. A higher L/A ratio is weakly associated with increased survival (odds ratio, OR = 1.001, p = 0.047, 95% confidence interval CI [1.000, 1.002]) [11]. The L/A ratio is a reliable prognostic factor independent of initial lactate and hepatic-renal function [12]. It has been proposed as a predictor of short and long-term mortality in critically ill patients with heart failure [13]. Furthermore, the L/A ratio is a better 30-day mortality predictor than lactate in hospitalized sepsis patients, although not in those with septic shock [14]. Patients with an L/A ratio >0.15 had greater long-term mortality [15].

We report a retrospective case series study that included 490 patients diagnosed with sepsis or septic shock secondary to SARS-CoV-2 pneumonia. Demographical data, prognostic scales, and biological markers were recorded. The primary outcome measure was the need for mechanical ventilation and 30-day mortality. The main objective was to evaluate the distribution of mortality between sepsis and septic shock patients and identify contributing factors (e.g., serum lactate, lactate/albumin ratio, C-reactive protein, and platelet levels) associated with 30-day mortality among COVID-19 patients. The secondary objectives were to study the factors associated with mechanical ventilation and assess the optimal cut-off threshold among these factors that can differentiate survivors from non-survivors. To the best of our knowledge, this study is the first to explore the predictive value of the L/A ratio for in-hospital mortality and disease severity. A high L/A ratio appears to predict higher odds of mortality and differentiate critical patients from mild or severe COVID-19 patients.

## 2 Methods

### 2.1 Study population

A longitudinal, observational, and retrospective study was conducted, including case reports of patients with pneumonia secondary to a laboratory-confirmed SARS-CoV-2 diagnosis (i.e., polymerase chain reaction) at the Department of Internal Medicine in two regional hospitals (Hospital General Regional No. 58 IMSS and Hospital General de Zone No. 21 de León, León, Mexico) from June 2020 through March 2021. Our findings are reported following the STROBE guidelines for cross-sectional studies.

The inclusion criteria were age >18 years, both sexes, positive PCR for SARS-CoV-2 virus, diagnosis of sepsis or septic shock, hospitalization at the Emergency Department less than 24 h, and patients with 30-day follow-up. The exclusion criteria comprised the diagnosis of hematologic or solid neoplasm and hepatic cirrhosis diagnosis. The elimination criteria included incomplete 30-day follow-up, loss of variables of interest, diagnosis of septic and another type of shock (i.e., hypovolemic, cardiogenic, anaphylactic, or neurogenic shock), sustaining an ischemic cerebrovascular event, and readmission to the ICU. No patients with bacterial sepsis or septic shock were included.

### 2.2 Clinical evaluation

Upon ICU admission, a comprehensive full blood and biochemistry workup was requested and extracted for our analysis. Patients were categorized based on the severity of their sepsis, assessed through chronic health status via comorbidity evaluation using the Charlson comorbidity index, the Sequential Organ Failure Assessment (SOFA), and the Acute Physiology and Chronic Health Evaluation (APACHE IV).

Sepsis was defined as a syndrome involving infection-induced physiological, pathological, and biochemical abnormalities [2]. The Sepsis-3 definition for septic shock was employed, which refers to sepsis characterized by persistent hypotension requiring vasopressor agents to maintain the mean arterial blood pressure ≥65 mmHg and lactate ≥180 mg/L (2 mmol/L) despite adequate fluid resuscitation [2].

### 2.3 Laboratory analysis

Hypoalbuminemia was defined as an albumin level <30 g/L or 3.0 g/dL [16]. The L/A ratio was calculated by dividing serum lactate (in mmol/L) by serum albumin (in g/dL). Demographical variables such as sex, age, and body mass index (BMI), as well as clinical variables such as intra-hospital stay, albumin, lactate, comorbidities, heart failure, acute kidney failure, acute respiratory failure, and therapeutic management (e.g., use of vasopressors and inotropes, renal replacement therapy, steroid use, mechanical ventilation) were obtained from the medical file. Recruitment bias was mitigated by enrolling consecutive patients. Upon medical admission, the patient or a family member signed an informed consent permitting the use of their medical file information for didactic, research, and publication purposes. This study was approved by the Institutional Review Board (Comité de Ética en Investigación 10058, with approval number CONBIOETICA 11 CEI 004 20190709, approved on June 30th, 2020) of the two participating institutions. Abiding by the Declaration of Helsinki, patient anonymity was guaranteed.

### 2.4 Statistical analysis

Statistical analysis was conducted using SPSS 26 (SPSS Inc., Chicago, IL). The study size was determined by previously published reports. Data were screened for outliers and adherence to normality assumptions. The normality of continuous variables (age, BMI, length of hospital stay, CCI, SOFA, APACHE IV, platelet count, serum C-reactive protein, serum lactate, serum albumin, L/A ratio) was assessed with the Shapiro-Wilk normality test and visually inspected through histograms and Q-Q plots.

Demographical and clinical factors are presented as proportions and percentages. For normally distributed variables reported in this study (mean, standard deviation, and range) were reported, while for non-parametric variables, central tendency measures included median, interquartile range (IQR), and range. Levene's test for equality of variances was employed to assess homoscedasticity. Due to different the sample sizes in the two independent groups assessed (n = 337 vs. n = 153 for sepsis and septic shock, respectively), the Welch and Brown-Forsythe t-tests were used to compare normally distributed continuous variables (age, BMI, and serum albumin).

The Mann-Whitney *U* test was applied for statistical inference between sepsis and septic shock groups for the non-parametric continuous variables (length of hospital stay, CCI, SOFA, APACHE IV, platelet count, C-reactive protein, serum lactate, and L/A ratio). The threshold for statistical significance was set at p ≤ .0038 after a Bonferroni correction for multiple comparisons.

### 2.5 Survival model

The Kaplan-Meier method was employed to calculate survival distributions, and the Gehan-Breslow-Wilcoxon method was used to compare the equality of survival distributions, giving more weight to deaths at early time points. A multivariable Cox proportional hazard model was utilized to assess the association between platelet count levels upon ICU admission and case fatality rate at 30 days.

The multiple Cox regression model with backward stepwise elimination included the following covariates: sex, age, BMI, APACHE score, SOFA score, Charlson comorbidity index, platelet count, C-reactive protein, serum lactate, serum albumin, L/A ratio, and two interaction variables (APACHE_score*SOFA_score*Charlson_comorbidity_index, albumin*lactate*LA_ ratio). Equality of survival times, pairwise comparisons (between-group comparisons), and statistical significance were assessed, with a significance level set at p ≤ .05.

The Omnibus Test of Model Coefficients was employed to evaluate overall model fitness and changes between models. The −2 log-likelihood statistic (–2LL) determined if the predictor contributed to the overall model. R_L_^2^ or Hosmer-Lemeshow R^2^ was computed using (–2LL_baseline_) – (–2LL_new_)/–2LL_baseline_.

Receiver operating characteristic (ROC) curves were generated for biomarkers to compare their diagnostic accuracy in predicting two time-to-event outcomes (need for mechanical intubation and mortality) based on their respective area under the curve (AUC). Optimal cut-off points for each biomarker, capable of discriminating statistically significant negative outcomes (need for mechanical intubation or mortality), were obtained using AUC, sensitivity, and specificity.

## 3 Results

### 3.1 Demographical, sepsis severity, and biomarker population description

A total of 490 patients were included in the analysis, with 286 males (58.4%). The mean age was 60.47 ± 14.62 years (range, 18–97), and the mean BMI was 28.50 ± 2.99 (range, 19.60–40.40). The median length of hospitalization for the entire population was 10 days (Interquartile range, IQR, 7–14 days; range, 1–62).

No statistically significant differences were observed between sexes for age, BMI, and length of hospitalization. Sepsis severity measures for all patients included in this study were a Charlson Comorbidity Index (CCI) median of 2 (IQR, 1–3 points; range, 0–8), Sequential Organ Failure Assessment (SOFA) median of 2 (IQR, 2–5 points; range, 1–15), and a median Acute Physiology and Chronic Health Evaluation (APACHE) IV score of 26 (IQR, 18–46.25 points; range, 6–80).

The biomarkers included in this study were platelet count (median, 243,000 per microliter; IQR, 200,000–325,250; range, 80,000–758,000), C-reactive protein levels (median, 15 mg/L; IQR, 10–32; range, 0.5–614), serum lactate levels (median, 1.00 mmol/L; IQR, 1.00–2.50; range, 0.40–5.00), serum albumin levels (median, 3.01 g/dL; IQR, 2.7–3.7; range, 1.5–4.5) and L/A ratio (median, 0.33; IQR, 0.23–0.90; range, 0.1–2.5) (see Table 1).

**Table 1:** Patient characteristics.

Variables	Overall (n = 490)	Sepsis (n = 337)	Septic Shock (n = 153)	*P*
Sex (male, %)	286 (58.4)	197 (58.5)	89 (58.2)	0.952
Age (years; SD, range)	60.46 (±14.63; 18–97)	58.79 (±15.19; 18–97)	64.16 (±12.61; 29–89)	≤0.001
Body-mass index (mean; SD, range)	28.50 (±2.99; 19.6–40.4)	28.48 ± 3.02	28.53 ± 2.92	0.882
Charlson comorbidity index (median; IQR, range)	2 (1–3; 0–8)	2 (1–3; 0–8)	2 (1–3; 0–8)	≤0.001
SOFA	2 (2–5; 1–15)	2 (2-2; 1–10)	8 (5–10; 2–15)	≤0.001
APACHE IV	26 (18–46.25; 6–80)	20 (18–26.5; 10–67)	50 (45–59; 6–80)	≤0.001
Platelet count	243 x 10^3^ (200–325.25; 80–758)	245 x 10^3^ (210–339.5; 80–758)	240 x 10^3^ (154–288.5; 80–673)	0.003
C-reactive protein	15 (10–32; 0.5–614)	14 (8–21.6; 0.5–603)	24 (15–35.9; 2.3–614)	≤0.001
Lactate	1.0 (1.0–2.5; 0.4–5.0)	1.0 (1.0–1.0; 0.4–4.5)	3.0 (2.5–3.5; 0.5–5.0)	≤0.001
Albumin	30.1 (27–37; 15–45)	35.0 (30–38; 18–45)	26.0 (25–28; 15–40)	≤0.001
Lactate to albumin ratio	0.33 (0.26–0.91; 0.1–2.5)	0.28 (0.25–0.33; 0.1–2.25)	1.12 (0.93–1.35; 0.2–2.5)	≤0.001
Length of hospital stay	10.0 (7–14; 1-62)	12 (8–15; 1–62)	9.0 (5–13; 2–36)	≤0.001

Data are shown as the median with interquartile range (IQR) and range, unless specified. SD: standard deviation. Organ dysfunction was defined as a SOFA score of two or more. SOFA, sepsis-related organ failure assessment; APACHE indicates acute physiology and chronic health evaluation.

### 3.2 Sepsis and septic shock group differences

#### 3.2.1 Demographical differences

**Fig. 1:**
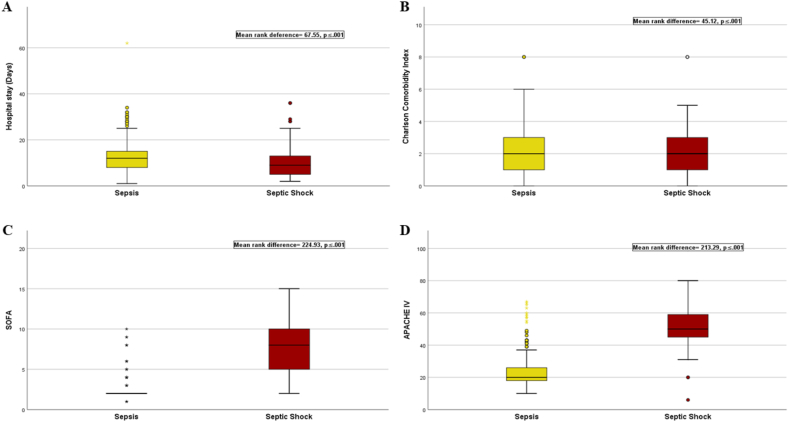
Sepsis severity according to clinical outcomes.

Sepsis and septic shock were assessed using the Sepsis-3 definition, resulting in 337 patients diagnosed with sepsis (71.8%) and 153 patients diagnosed with septic shock. Patients with septic shock were found to be older than patients with sepsis (mean age, septic shock = 64.16 ± 12.61, sepsis = 58.79 ± 15.17; mean difference, 5.37 years, p ≤ 0.001, 95%CI [2.60, 8.13]). No statistically significant differences were observed in BMI between the two groups (mean difference = 0.04, p = 0.882, 95%CI [−0.62, 0.53]). Additionally, patients with sepsis exhibited a longer length of hospital stay compared to those with septic shock (sepsis, median = 12 days, IQR 8–15, range, 1–62; septic shock, median = 9 days, IQR, 5–13, range 2–36; mean rank deference = 67.55, p ≤ 0.001) (see Fig. 1a).

#### 3.2.2 Sepsis severity differences

Patients with septic shock exhibited a higher burden of comorbidities, as reflected by the CCI (sepsis, median = 2 points, IQR, 1–3, range, 0–8; septic shock, median = 2 points, IQR, 1–3, range 0–8; mean rank difference = −45.12, p ≤ 0.001). Moreover, they demonstrated a higher level of organ failure assessed by the SOFA (sepsis, median = 2 points, IQR 2-2, range, 1–11; septic shock, median = 8 points, IQR, 5–10, range, 2–15; mean rank difference = −224.93, p ≤ 0.001). Furthermore, patients with septic shock had higher APACHE IV scores compared to patients with patients with sepsis (sepsis, median = 20, IQR, 18–29, range, 10–70; septic shock, median = 50 points, IQR, 50–59, range, 6–80; mean rank difference = −213.29, p ≤ 0.001) (see Fig. 1b–d).

#### 3.2.3 Biomarker differences

Among the biomarkers assessed in this study, C-reactive protein levels were higher in patients with septic shock compared to sepsis (sepsis, median = 14.0 mg/L, IQR, 8–22, range, 0.5–614; septic shock, median = 24.0 mg/L, IQR, 15–38.85, range, 2.3–200; mean rank difference = −123.19, p = 0.001). Similarly, serum lactate levels were elevated in septic shock compared to sepsis (sepsis, median = 1.00 mmol/L, IQR, 1.00–1.00, range, 0.4–3.7; septic shock, median = 3.00 mmol/L, IQR, 2.50–3.50, range, 2–5; mean rank difference = −236.72, p ≤ 0.001), as well as the L/A ratio (sepsis, median = 0.28, IQR, 0.25–0.33, range, 0.1–2.25; septic shock, median = 1.12, IQR, 0.93–1.35, range, 0.2–2.5; mean rank difference = −240.18, p ≤ 0.001).

**Fig. 2:**
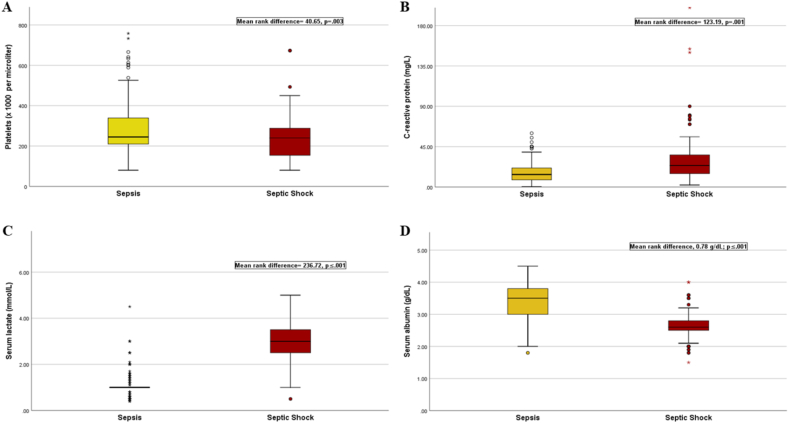
Sepsis severity according to biomarkers.

On the other hand, patients with sepsis had higher serum albumin levels compared to those with septic shock (median, 3.5 g/dL, IQR, 30–38, range 18–45; mean rank difference, 0.78 g/dL; p ≤ 0.001; 95%CI [6.92, 8.61]). Platelet levels were also greater in sepsis compared to septic shock (sepsis, median = 245,000 per microliter, IQR, 210,000–339,500, range 80,000–758,000; septic shock, median = 240,000 per microliter, IQR, 154,000–288,500, range, 80,000–673,000; mean rank difference = 40.65, p = 0.003; 95%CI [27.32, 60.33]) (see Fig. 2a–d).

#### 3.2.4 Outcome assessment: mechanical ventilation and mortality

**Fig. 3:**
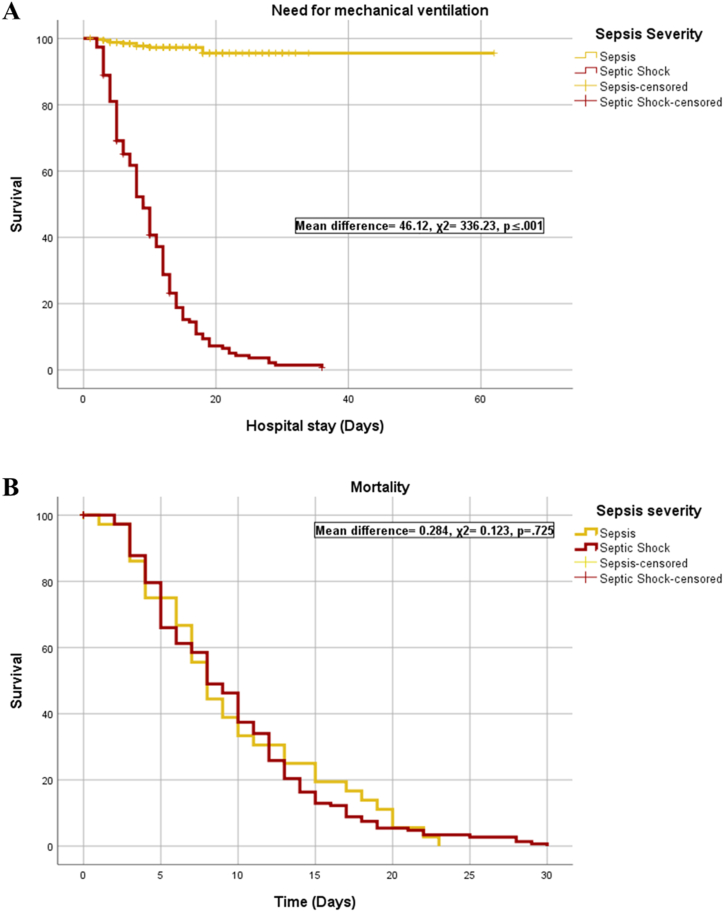
Time-to-event analyses for sepsis severity and ROC analysis.

After assessing demographic factors, sepsis severity, and biomarkers between sepsis and septic shock patients with COVID-19, we conducted time-to-event analyses to evaluate the need for mechanical ventilation (n = 154) and mortality (n = 183). Patients with septic shock were found to have a higher likelihood of requiring invasive ventilator support compared to patients with sepsis (mean difference = −46.12, χ^2^ = 336.23, p ≤ 0.001) (see Fig. 3a). However, the distribution of mortality was similar between the sepsis and septic shock patient groups (mean difference = −0.284, χ^2^ = 0.123, p = 0.725; see Fig. 3b).

Multiple variate analysis was performed to identify factors associated with a higher risk of invasive mechanical intubation in patients with septic shock. Three factors were found to be associated with a higher risk of intubation: APACHE IV (OR: 1.027, p = 0.005, 95% CI [1.008, 1.046]), serum albumin levels (OR: 0.805, p ≤ 0.001, 95% CI [0.732, 0.885]), and L/A ratio (OR: 0.001, p = 0.001, 95% CI [0.001, 0.001]). In contrast, sex, age, BMI, CCI score, SOFA score, platelet count, serum C-reactive protein levels, serum lactate levels, and the two interaction variables (sepsis severity and biomarker interaction variables) previously described were not statistically relevant (see Table 2).

**Table 2:** COX regression model coefficients and effect sizes.

	Mechanical ventilation (n = 154)				95% CI for Odds Ratio			Pseudo-R^2^
	Variable	χ^2^	p	b	Lower	Odds	Upper	H&L
Demographical	Sex	0.087	0.768	0.051	0.752	1.052	1.472	≤0.001
	Age	0.044	0.834	0.001	0.988	1.001	1.016	0.001
	Body-mass index	0.301	0.583	0.017	0.958	1.017	1.079	0.001
Sepsis severity scales	Charlson comorbidity index	2.497	0.114	0.190	0.955	1.210	1.532	0.005
	SOFA	5.565	0.018	0.129	1.022	1.138	1.267	0.02
	**APACHE IV**	**7.731**	0**.005**	0**.026**	**1.008**	**1.027**	**1.046**	0**.02**
	CCI*SOFA*APACHE IV	6.415	0.011	−0.001	0.999	0.999	1.000	0.01
Biomarkers	Platelet count	1.343	0.247	−0.001	0.997	0.999	1.001	0.005
	C-reactive protein	0.496	0.481	0.002	0.997	1.002	1.007	≤0.001
	Lactate	5.083	0.024	1.739	1.255	5.693	25.821	0.041
	**Albumin**	**20.027**	**≤**0**.001**	**−**0**.216**	0**.732**	0**.805**	0**.885**	0**.039**
	**Lactate to albumin ratio**	**11.740**	0**.001**	**−40.174**	0**.001**	0**.001**	0**.001**	0**.04**
	Lactate*albumin*L/A ratio	0.087	0.768	−0.031	0.786	0.969	1.195	≤0.001

CI: confidence interval. χ^2^: Wald test. Beta value refers to the measure of the modeled effect that reflects the parameter estimate. All reported p-values are corrected with a Bonferroni correction for multiple comparisons. H&L: Hosmer & Lemeshow R^2^. CCI: Charlson comorbidity index. APACHE IV: Acute Physiology and Chronic Health Evaluation. SOFA: Sequential Organ Failure Assessment. L/A ratio: lactate to albumin ratio.

#### 3.2.5 ROC analysis

**Fig. 4:**
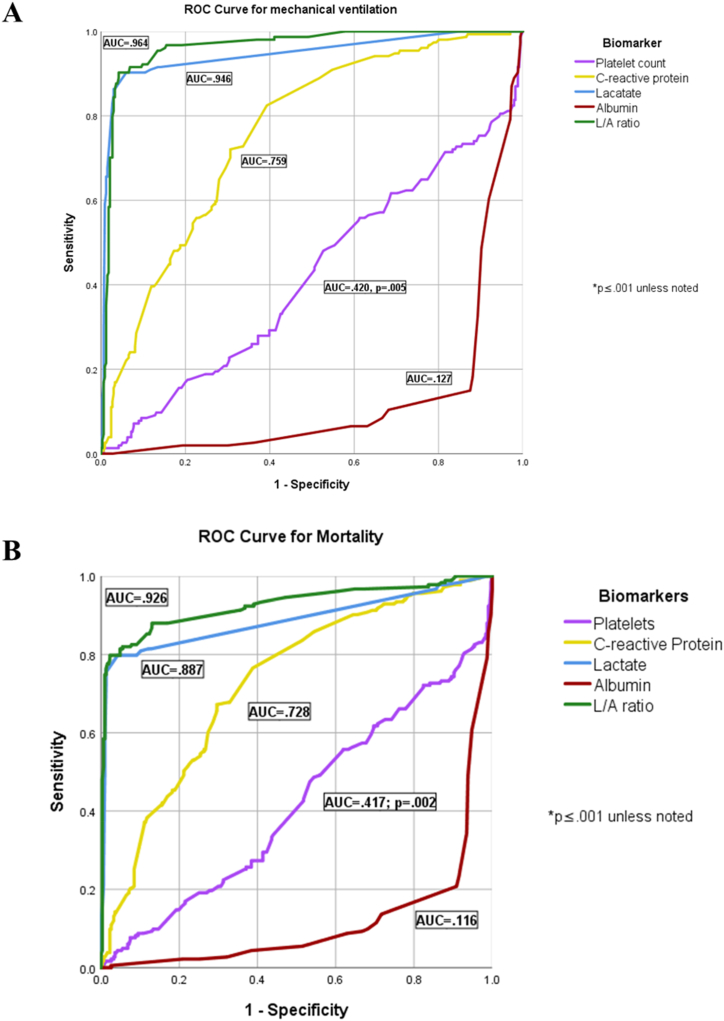
Biomarker ROC curves for mechanical ventilation and mortality.

A receiver operating characteristic analysis was conducted to identify the optimal cut-off points for biomarkers capable of discriminating between the need for invasive mechanical ventilation and mortality (see Fig. 4a and b). Both the L/A ratio and serum lactate levels exhibited the highest diagnostic accuracy for predicting the need for mechanical ventilation (AUC, 0.964 and 0.946, respectively) and mortality (AUC, 0.926 and 0.887, respectively). C-reactive protein demonstrated modest diagnostic accuracy for both the need for mechanical ventilation and mortality (AUC of 0.759 and 0.728, respectively). In contrast, platelet count and serum albumin levels showed the least diagnostic accuracy for these adverse outcome measures (platelets, 0.420 and 0.417, for mechanical ventilation and mortality, respectively; serum albumin levels, 0.127 and 0.116, for mechanical ventilation and mortality, respectively).

The optimal L/A ratio cut-off point for discriminating between the need for mechanical ventilation and mortality was 0.413 for both outcomes. This determination was based on the furthest away point, corresponding to the coordinates (0.903, 0.119) for mechanical ventilation and (0.809, 0.101) for mortality. For serum lactate levels, the optimal cut-off points were identified as 1.65 mmol/L (coordinates, 0.870, 0.077) for mechanical ventilation and 1.45 mmol/L (coordinates, 0.770, 0.091) for mortality.

**Fig. 5:**
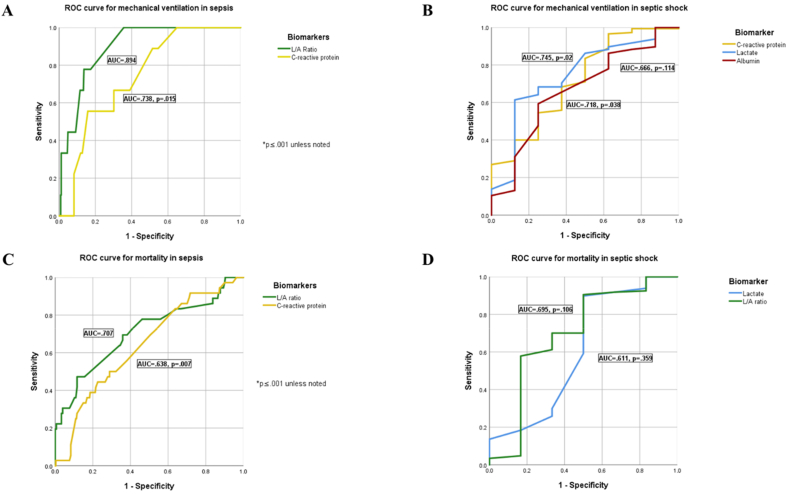
ROC for mechanical ventilation and mortality in sepsis and septic shock.

When considering patients with sepsis, only the L/A ratio and C-reactive protein levels demonstrated low diagnostic accuracy for predicting the need for mechanical ventilation (L/A ratio, AUC = 0.894, cut-off point = 0.344, (0.696, 0.185); C-reactive protein, AUC = 0.738, cut-off = 15.950, (0.652, 0.325)) and the L/A ratio exhibited low diagnostic accuracy for predicting mortality (AUC = 0.707, cut-off point = 0.338, (0.520, 0.175)), while C-reactive protein had a low diagnostic accuracy with AUC = 0.638 (cut-off point = 15.45 (0.540, 0.318)) (see Fig. 5a and b). Among patients with septic shock, serum lactate levels showed moderate diagnostic accuracy for predicting patient intubation (AUC = 0.745, cut-off = 2.95, (0.641, 0.286)); while C-reactive protein and serum lactate exhibited low diagnostic accuracy (i.e., 0.718 and 0.666, respectively) (see Fig. 5c). Only the L/A ratio had low diagnostic accuracy for classifying mortality (AUC = 0.695, cut-off = 1.11, (0.609, 0.200)) (see Fig. 5d). The AUC and cut-off values for all the biomarkers are reported in Table 3.

**Table 3:** Area under the curve for ROC analyses.

		Need for mechanical ventilation (n = 154)			Mortality (n = 183)		
**Population**	**Biomarker**	**AUC**	**Cut-off**	**Coordinates**	**AUC**	**Cut-off**	**Coordinates**
**Overall**	Platelet count (per μL)	0.420	234,500	(0.565, 0.634)	0.417	240,500	(0.492, 0.560)
	C-reactive protein (mg/L)	0.759	15.95	(0.727, 0.330)	0.728	15.45	(0.678, 0.326)
	**Serum lactat**e (mmol/L)	0**.946**	**1.65**	**(**0**.870,** 0**.077)**	0**.887**	**1.45**	**(**0**.770,** 0**.091)**
	Serum albumin (g/dL)	0.127	2.85	(0.182, 0.881)	0.116	2.95	(0.208, 0.909)
	**L/A ratio**	0**.964**	0**.413**	**(**0**.903,** 0**.119)**	0**.926**	**0.413**	**(**0**.809,** 0**.101)**
**Sepsis**	Platelet count	0.531	242,500	(0.652, 0.526)	0.486	240,500	(0.560, 0.560)
	**C-reactive protein**	0**.738**	**15.95**	**(**0**.652,** 0**.325)**	0**.638**	**15.45**	**(**0**.540,** 0**.318)**
	Serum lactate	0.564	1.05	(0.217, 0.140)	0.546	1.15	(0.220, 0.126)
	Serum albumin	0.177	2.75	(0.348, 0.912)	0.205	2.75	(0.480, 0.940)
	**L/A ratio**	0**.894**	0**.344**	**(**0**.696,** 0**.185)**	0**.707**	0**.338**	**(**0**.520,** 0**.175)**
**Septic shock**	Platelet count	0.450	244,500	(0.405, 0.286)	0.342	239,000	(0.534, 0.600)
	**C-reactive protein**	0**.718**	**17.50**	**(**0**.687,** 0**.429)**	0.457	32.65	(0.406, 0.200)
	**Serum lactate**	0**.745**	**2.95**	**(**0**.641,** 0**.286)**	0.611	2.45	(0.910, 0.600)
	Serum albumin	0.666	25.5	(0.611, 0.143)	0.354	26.50	(0.466, 0.800)
	L/A ratio	0.442	1.183	(0.450, 0.429)	0.695	1.091	(0.609, 0.200)

AUC: area under the curve. ROC: receiver operating characteristic. L/A: Lactate to albumin.

## 4 Discussion

### 4.1 Main findings

In the case series, we investigate the association between three sepsis severity prognostic scales and five biomarkers associated with inflammation to two poor clinical outcomes: the need for invasive mechanical ventilation and mortality at 30 days. Notably, this study represents one of the first efforts to explore between-group biomarker differences among critically ill patients, specifically those with sepsis and septic shock, concerning the outcomes of mechanical ventilation requirement and 30-day mortality in COVID-19 patients.

In our study, sepsis patients exhibited higher serum albumin and platelet levels, while those with septic shock were characterized by advanced age, a shorter length of hospitalization, more comorbidities, and higher organ failure indices and APACHE scores. Additionally, C-reactive protein, serum lactate, and the L/A ratio were significantly elevated in patients with septic shock compared to those with sepsis. Although patients with septic shock were more likely to require invasive mechanical ventilation, their mortality distributions over time resembled those of patients with sepsis. Serum albumin levels and APACHE IV scores were identified as factors associated with a higher risk of intubation. Optimal cut-off values for lactate (1.45–1.65 mmol/L) and the L/A ratio (0.413) were obtained to discriminate between the need for mechanical ventilation and mortality.

### 4.2 Relation to the current literature

Recently, Gök and colleagues (2021) evaluated the L/A ratio in patients with COVID-19, reporting its superior diagnostic accuracy for predicting 30-day mortality compared to lactate and albumin alone [17]. In our study, we observed that patients with sepsis exhibited higher serum albumin and platelet levels than those with septic shock. The septic shock group, however, was characterized by advanced age, a shorter length of hospitalization, more comorbidities, a higher organ failure index, and an elevated APACHE score. Additionally, C-reactive protein, serum lactate, and L/A ratio were significantly higher among patients with septic shock than in patients with sepsis. While patients with septic shock were more likely to require invasive mechanical ventilation, their mortality distributions over time were like those of patients with sepsis.

Comparing albumin levels between survivors and non-survivors in patients with sepsis lower albumin levels (mean, 2.7 ± 0.7 g/dL versus 2.9 ± 0.6 g/dL), along with higher serum lactate levels (mean, 3.6 ± 3 mmol/L versus 2.1 ± 1.5 mmol/L), and a higher L/A ratio (mean 1.5 ± 1.4 versus 0.8 ± 0.6), all linked to higher mortality [18]. In patients with septic shock, a threshold of 1.4 mmol/L predicted multiple-organ dysfunction syndrome (MODS) and mortality [19]. The L/A ratio has shown moderate predictive value for mortality, regardless of initial lactate levels or the presence of kidney or liver failure [18].

Bou Chebl et al. (2020) investigated serum lactate levels and L/A ratio as predictors of in-hospital mortality in septic shock patients, yielding an AUC of 0.61 for lactate and an AUC of 0.67 for the L/A ratio. The L/A ratio demonstrated predictive value for in-hospital mortality (p < 0.001, OR 1.53, 95% CI [1.32, 1.78]) [20]. In contrast to undifferentiated septic shock, COVID-19 critical illness exhibited minimal evidence of systemic tissue hypoperfusion, cytokine release, endothelial injury, or microcirculatory flow impairment [21].

Gök and colleagues (2021) explored the L/A ratio as a predictor of 30-day intrahospital mortality among critically ill COVID-19 patients. Non-survivors exhibited higher lactate levels (2.77 vs. 1.73 mmol/L, p < 0.001) and lower albumin levels (2.73 vs. 2.95 g/dL, p < 0.001) compared to survivors. The L/A ratio had an AUC of 0.824 (p < 0.001), surpassing serum albumin levels (AUC = 0.644) and serum lactate levels (AUC = 0.795) [17]. Patients with an L/A ratio >0.60 upon ICU admission had organ failure and higher APACHE-II scores (p < 0.001), establishing it as an independent 30-day mortality risk factor (HR = 10.615, p < 0.001, 95% CI [5.673, 19.865]).

### 4.3 Limitations and future perspectives

The study has certain limitations that need consideration. Patients enrolled in the study had pre-existing conditions such as malnutrition, chronic kidney disease, and potential liver alterations before their SARS-CoV-2 infection. These conditions could impact the interpretation of the assessed biomarkers. Additionally, there was a limitation in the monitoring of albumin levels during hospitalization. The lack of frequent and sequential albumin measurements is noteworthy, as critically ill patients undergo distinct phases, marked by rapid albumin loss during deterioration and potential recovery or stabilization in albumin serum level [22]. Albumin kinetics vary among patients and are influenced by factors like hepatic disease, malignancy, kidney disease, and albumin transfusion [22].

Moreover, the study did not include relevant acute proinflammatory biomarkers such as IL-6, IL-1b, procalcitonin, troponin, and N-terminal pro-B-type natriuretic peptide [23,24]. Future investigations considering these aspects could provide a more comprehensive picture of the patient's condition and contribute to a more robust interpretation of the findings. Future analyses of diagnostic accuracy and prediction should consider more complex models, including stability analyses, numerical solutions of fractional order, and machine learning methods (e.g., Random Forest, Gradient Boosting, Adaptive Boosting, Stacking, XGBoost, and LightGBM). These methods are particularly valuable when dealing with complex, real-world problems, as they can enhance the generalization and robustness of models, often outperforming individual models.

## 5 Conclusion

The study findings highlight the potential utility of a composite assessment involving increased C-reactive protein, elevated serum lactate, and a high lactate/albumin (L/A) ratio for clinicians in identifying patients at risk of mechanical ventilation and mortality upon admission, particularly within the context of sepsis and septic shock associated with viral pneumonia. The proposed optimal cut-off values for lactate (1.45–1.65 mmol/L) and the L/A ratio (0.413) offer practical thresholds that may assist in prioritizing medical care for individuals with COVID-19 who face a heightened risk of mortality. These insights contribute valuable information for clinical decision-making and resource allocation in the management of critically ill patients.

## Data availability statement

The data that support the findings of this study are available on request from the corresponding author, JDM.

## CRediT authorship contribution statement

**O. Jiménez-Zarazúa:** Writing – original draft, Supervision, Project administration, Methodology, Investigation, Data curation, Conceptualization. **L.N. Vélez-Ramírez:** Writing – review & editing, Formal analysis, Data curation, Conceptualization. **J.D. Mondragón:** Writing – review & editing, Writing – original draft, Visualization, Supervision, Software, Resources, Methodology, Funding acquisition, Formal analysis, Data curation, Conceptualization.

## Declaration of competing interest

The authors declare that they have no known competing financial interests or personal relationships that could have appeared to influence the work reported in this paper.
