# Supplementary material for: Biomarkers and sepsis severity as predictors of mechanical ventilation and mortality in COVID-19
Source: Heliyon. 2024 Mar 25;10(7):e28521. doi: 10.1016/j.heliyon.2024.e28521 (PMC10990852; doi:10.1016/j.heliyon.2024.e28521)
Supplement: Multimedia component 1 [file mmc1.docx]

**Appendix**

**Supplemental Table 1. Biomarker performance and cut-offs for mechanical ventilation**

| **Coordinates of the Curve** | | | |
| --- | --- | --- | --- |
| Test Result Variable(s) | Positive if Greater Than or Equal To^a^ | Sensitivity | 1 - Specificity |
| Platelets | 79.00 | 1.000 | 1.000 |
|  | 82.50 | .987 | .994 |
|  | 86.50 | .981 | .994 |
|  | 94.00 | .968 | .994 |
|  | 104.00 | .916 | .991 |
|  | 109.00 | .916 | .988 |
|  | 111.00 | .870 | .988 |
|  | 112.50 | .870 | .985 |
|  | 116.50 | .870 | .982 |
|  | 122.00 | .838 | .982 |
|  | 127.00 | .838 | .979 |
|  | 133.00 | .831 | .979 |
|  | 136.50 | .825 | .979 |
|  | 138.50 | .825 | .976 |
|  | 141.50 | .812 | .970 |
|  | 143.50 | .812 | .967 |
|  | 144.50 | .812 | .964 |
|  | 145.50 | .805 | .952 |
|  | 146.50 | .805 | .949 |
|  | 147.50 | .805 | .946 |
|  | 149.00 | .799 | .943 |
|  | 150.50 | .786 | .926 |
|  | 152.00 | .779 | .923 |
|  | 153.50 | .766 | .920 |
|  | 154.50 | .753 | .911 |
|  | 155.50 | .753 | .896 |
|  | 156.50 | .747 | .893 |
|  | 157.50 | .740 | .890 |
|  | 158.50 | .740 | .887 |
|  | 159.50 | .740 | .884 |
|  | 160.50 | .734 | .884 |
|  | 161.50 | .734 | .881 |
|  | 163.50 | .734 | .875 |
|  | 166.00 | .734 | .872 |
|  | 168.50 | .734 | .869 |
|  | 171.00 | .734 | .857 |
|  | 172.50 | .727 | .854 |
|  | 174.50 | .727 | .851 |
|  | 181.00 | .727 | .842 |
|  | 186.50 | .721 | .839 |
|  | 188.50 | .721 | .833 |
|  | 191.50 | .714 | .833 |
|  | 193.50 | .714 | .830 |
|  | 195.50 | .714 | .818 |
|  | 198.50 | .714 | .815 |
|  | 200.50 | .649 | .774 |
|  | 201.50 | .649 | .768 |
|  | 203.00 | .649 | .765 |
|  | 207.00 | .649 | .759 |
|  | 211.00 | .623 | .738 |
|  | 215.00 | .623 | .735 |
|  | 219.00 | .623 | .729 |
|  | 220.50 | .623 | .723 |
|  | 222.00 | .617 | .702 |
|  | 224.00 | .617 | .699 |
|  | 225.50 | .617 | .696 |
|  | 226.50 | .617 | .690 |
|  | 228.50 | .617 | .688 |
|  | 230.50 | .597 | .682 |
|  | 232.00 | .571 | .670 |
|  | 233.50 | .571 | .661 |
|  | 234.50 | .565 | .634 |
|  | 235.50 | .558 | .625 |
|  | 236.50 | .558 | .622 |
|  | 237.50 | .558 | .613 |
|  | 239.00 | .552 | .610 |
|  | 240.50 | .494 | .554 |
|  | 241.50 | .487 | .539 |
|  | 242.50 | .481 | .527 |
|  | 243.50 | .442 | .506 |
|  | 244.50 | .435 | .506 |
|  | 245.50 | .338 | .432 |
|  | 247.50 | .331 | .429 |
|  | 249.50 | .331 | .426 |
|  | 253.00 | .292 | .414 |
|  | 256.50 | .292 | .411 |
|  | 261.50 | .292 | .405 |
|  | 269.00 | .292 | .402 |
|  | 273.00 | .292 | .399 |
|  | 274.50 | .286 | .399 |
|  | 275.50 | .279 | .399 |
|  | 276.50 | .279 | .396 |
|  | 278.50 | .279 | .390 |
|  | 280.50 | .279 | .387 |
|  | 281.50 | .279 | .384 |
|  | 282.50 | .279 | .381 |
|  | 284.00 | .279 | .378 |
|  | 285.50 | .279 | .375 |
|  | 287.00 | .279 | .372 |
|  | 288.50 | .266 | .372 |
|  | 289.50 | .260 | .372 |
|  | 292.00 | .260 | .360 |
|  | 295.00 | .260 | .357 |
|  | 298.00 | .253 | .357 |
|  | 304.50 | .227 | .304 |
|  | 309.50 | .221 | .304 |
|  | 311.00 | .208 | .298 |
|  | 315.50 | .208 | .295 |
|  | 320.00 | .208 | .292 |
|  | 323.00 | .195 | .277 |
|  | 325.50 | .195 | .274 |
|  | 327.00 | .195 | .271 |
|  | 328.50 | .188 | .265 |
|  | 330.00 | .188 | .262 |
|  | 331.50 | .188 | .259 |
|  | 333.00 | .188 | .256 |
|  | 334.50 | .188 | .253 |
|  | 337.00 | .188 | .250 |
|  | 339.50 | .188 | .247 |
|  | 340.50 | .175 | .208 |
|  | 341.50 | .175 | .205 |
|  | 342.50 | .169 | .199 |
|  | 343.50 | .156 | .190 |
|  | 344.50 | .156 | .185 |
|  | 345.50 | .097 | .143 |
|  | 347.50 | .097 | .131 |
|  | 349.50 | .091 | .128 |
|  | 351.00 | .084 | .107 |
|  | 352.50 | .084 | .104 |
|  | 353.50 | .084 | .101 |
|  | 354.50 | .084 | .098 |
|  | 357.50 | .084 | .095 |
|  | 360.50 | .078 | .095 |
|  | 362.50 | .078 | .092 |
|  | 365.00 | .078 | .089 |
|  | 368.00 | .071 | .089 |
|  | 371.00 | .071 | .086 |
|  | 375.00 | .071 | .083 |
|  | 384.00 | .071 | .080 |
|  | 392.00 | .071 | .077 |
|  | 397.00 | .065 | .077 |
|  | 402.50 | .052 | .074 |
|  | 406.50 | .052 | .071 |
|  | 409.50 | .045 | .071 |
|  | 417.50 | .039 | .071 |
|  | 427.50 | .039 | .068 |
|  | 432.00 | .039 | .065 |
|  | 434.00 | .039 | .063 |
|  | 437.50 | .032 | .063 |
|  | 443.50 | .026 | .060 |
|  | 448.50 | .026 | .051 |
|  | 458.50 | .019 | .048 |
|  | 476.50 | .019 | .045 |
|  | 489.50 | .019 | .042 |
|  | 494.50 | .013 | .042 |
|  | 498.00 | .013 | .039 |
|  | 504.50 | .013 | .036 |
|  | 517.50 | .013 | .033 |
|  | 532.00 | .013 | .030 |
|  | 563.50 | .013 | .027 |
|  | 594.50 | .013 | .024 |
|  | 602.00 | .013 | .021 |
|  | 608.50 | .013 | .018 |
|  | 623.00 | .013 | .015 |
|  | 637.00 | .013 | .012 |
|  | 653.50 | .013 | .009 |
|  | 669.50 | .013 | .006 |
|  | 703.00 | .000 | .006 |
|  | 745.50 | .000 | .003 |
|  | 759.00 | .000 | .000 |
| C-reactive protein | .5000 | 1.000 | 1.000 |
|  | .5500 | 1.000 | .997 |
|  | .6500 | 1.000 | .994 |
|  | .8500 | 1.000 | .991 |
|  | 1.2000 | 1.000 | .988 |
|  | 1.6500 | 1.000 | .985 |
|  | 1.9500 | 1.000 | .979 |
|  | 2.0500 | 1.000 | .973 |
|  | 2.2000 | 1.000 | .970 |
|  | 2.4000 | .994 | .970 |
|  | 2.5500 | .994 | .967 |
|  | 2.7000 | .994 | .946 |
|  | 2.9000 | .994 | .938 |
|  | 3.1500 | .994 | .923 |
|  | 3.3500 | .994 | .920 |
|  | 3.4500 | .994 | .917 |
|  | 3.6000 | .994 | .914 |
|  | 3.8000 | .994 | .911 |
|  | 3.9500 | .994 | .902 |
|  | 4.1000 | .994 | .878 |
|  | 4.2500 | .994 | .875 |
|  | 4.4000 | .994 | .869 |
|  | 4.7500 | .987 | .866 |
|  | 5.1500 | .981 | .821 |
|  | 5.3500 | .981 | .813 |
|  | 5.4500 | .981 | .810 |
|  | 5.6000 | .981 | .801 |
|  | 5.8000 | .974 | .795 |
|  | 5.9500 | .974 | .789 |
|  | 6.0500 | .968 | .780 |
|  | 6.3000 | .968 | .777 |
|  | 6.5500 | .961 | .774 |
|  | 6.8000 | .961 | .771 |
|  | 7.0500 | .961 | .765 |
|  | 7.3000 | .961 | .762 |
|  | 7.6500 | .961 | .759 |
|  | 7.9000 | .955 | .753 |
|  | 8.1500 | .955 | .732 |
|  | 8.3500 | .955 | .729 |
|  | 8.4500 | .955 | .720 |
|  | 8.5500 | .955 | .708 |
|  | 8.7500 | .948 | .705 |
|  | 8.9500 | .948 | .696 |
|  | 9.5000 | .942 | .682 |
|  | 10.5000 | .942 | .658 |
|  | 11.5000 | .942 | .649 |
|  | 12.5000 | .909 | .548 |
|  | 13.5000 | .890 | .518 |
|  | 14.5000 | .825 | .393 |
|  | 15.4500 | .727 | .336 |
|  | 15.9500 | .727 | .333 |
|  | 16.1000 | .721 | .307 |
|  | 16.2500 | .714 | .307 |
|  | 16.6500 | .701 | .307 |
|  | 17.5000 | .675 | .295 |
|  | 18.5000 | .649 | .280 |
|  | 19.1000 | .597 | .274 |
|  | 19.3500 | .597 | .271 |
|  | 19.7000 | .591 | .271 |
|  | 19.9500 | .591 | .268 |
|  | 20.2500 | .584 | .262 |
|  | 20.7500 | .578 | .262 |
|  | 21.1000 | .565 | .247 |
|  | 21.6000 | .565 | .244 |
|  | 22.1500 | .558 | .232 |
|  | 22.6500 | .558 | .226 |
|  | 23.4000 | .545 | .217 |
|  | 23.9000 | .532 | .217 |
|  | 24.5000 | .513 | .208 |
|  | 25.2500 | .494 | .199 |
|  | 25.5500 | .494 | .193 |
|  | 25.8000 | .494 | .188 |
|  | 26.5000 | .487 | .188 |
|  | 27.4500 | .481 | .182 |
|  | 27.9500 | .481 | .173 |
|  | 28.5000 | .468 | .167 |
|  | 29.5000 | .461 | .164 |
|  | 30.5000 | .455 | .164 |
|  | 31.4500 | .448 | .164 |
|  | 31.9500 | .448 | .161 |
|  | 32.0500 | .403 | .131 |
|  | 32.5500 | .396 | .125 |
|  | 33.1500 | .396 | .122 |
|  | 33.6500 | .396 | .119 |
|  | 34.5000 | .286 | .083 |
|  | 35.4000 | .253 | .080 |
|  | 35.9000 | .247 | .080 |
|  | 36.5000 | .240 | .080 |
|  | 37.1500 | .240 | .071 |
|  | 38.1500 | .240 | .068 |
|  | 39.5000 | .234 | .065 |
|  | 41.5000 | .227 | .065 |
|  | 44.0000 | .221 | .060 |
|  | 45.1000 | .169 | .036 |
|  | 45.6000 | .169 | .033 |
|  | 48.0000 | .162 | .033 |
|  | 51.0000 | .156 | .030 |
|  | 53.0000 | .149 | .030 |
|  | 54.5000 | .143 | .030 |
|  | 55.5000 | .123 | .027 |
|  | 58.0000 | .110 | .027 |
|  | 65.0000 | .110 | .024 |
|  | 72.5000 | .097 | .024 |
|  | 75.5000 | .091 | .024 |
|  | 77.5000 | .084 | .024 |
|  | 79.5000 | .078 | .024 |
|  | 85.0000 | .065 | .024 |
|  | 120.0000 | .058 | .024 |
|  | 152.0000 | .052 | .024 |
|  | 177.0000 | .045 | .024 |
|  | 216.0000 | .039 | .024 |
|  | 260.0000 | .039 | .021 |
|  | 294.0000 | .039 | .018 |
|  | 320.0000 | .026 | .012 |
|  | 345.0000 | .026 | .009 |
|  | 377.5000 | .019 | .009 |
|  | 407.0000 | .013 | .009 |
|  | 429.5000 | .013 | .006 |
|  | 525.5000 | .006 | .006 |
|  | 602.0000 | .006 | .003 |
|  | 608.5000 | .006 | .000 |
|  | 615.0000 | .000 | .000 |
| Lactate | .4000 | 1.000 | 1.000 |
|  | .4500 | 1.000 | .988 |
|  | .5500 | 1.000 | .914 |
|  | .6500 | 1.000 | .878 |
|  | .7500 | 1.000 | .863 |
|  | .9000 | 1.000 | .851 |
|  | 1.0500 | .916 | .140 |
|  | 1.1500 | .916 | .134 |
|  | 1.2500 | .909 | .116 |
|  | 1.3500 | .903 | .104 |
|  | 1.4500 | .903 | .089 |
|  | 1.5500 | .903 | .074 |
|  | 1.6500 | .903 | .063 |
|  | 1.8500 | .903 | .060 |
|  | 2.0500 | .864 | .033 |
|  | 2.2000 | .864 | .030 |
|  | 2.3500 | .851 | .030 |
|  | 2.4500 | .831 | .027 |
|  | 2.5500 | .675 | .015 |
|  | 2.6500 | .656 | .015 |
|  | 2.7500 | .656 | .012 |
|  | 2.8500 | .617 | .012 |
|  | 2.9500 | .591 | .009 |
|  | 3.1000 | .292 | .006 |
|  | 3.3000 | .273 | .006 |
|  | 3.4500 | .253 | .006 |
|  | 3.5500 | .175 | .006 |
|  | 3.6500 | .130 | .003 |
|  | 3.8500 | .123 | .003 |
|  | 4.2500 | .045 | .003 |
|  | 4.5500 | .032 | .000 |
|  | 4.8000 | .006 | .000 |
|  | 6.0000 | .000 | .000 |
| Albumin | .5000 | 1.000 | 1.000 |
|  | 1.6500 | 1.000 | .997 |
|  | 1.8500 | .987 | .994 |
|  | 1.9500 | .974 | .994 |
|  | 2.0500 | .903 | .988 |
|  | 2.2000 | .890 | .979 |
|  | 2.3500 | .870 | .973 |
|  | 2.4500 | .792 | .970 |
|  | 2.5500 | .604 | .920 |
|  | 2.6500 | .487 | .902 |
|  | 2.7500 | .325 | .893 |
|  | 2.8500 | .182 | .881 |
|  | 2.9500 | .149 | .875 |
|  | 3.0500 | .104 | .682 |
|  | 3.1500 | .084 | .667 |
|  | 3.2500 | .071 | .643 |
|  | 3.3500 | .065 | .631 |
|  | 3.4500 | .065 | .592 |
|  | 3.5500 | .045 | .479 |
|  | 3.6500 | .026 | .363 |
|  | 3.7500 | .019 | .301 |
|  | 3.8500 | .019 | .232 |
|  | 3.9500 | .019 | .193 |
|  | 4.0500 | .000 | .027 |
|  | 4.1500 | .000 | .024 |
|  | 4.3000 | .000 | .015 |
|  | 4.4500 | .000 | .012 |
|  | 5.5000 | .000 | .000 |
| L/A ratio | .0900 | 1.000 | 1.000 |
|  | .1056 | 1.000 | .997 |
|  | .1181 | 1.000 | .994 |
|  | .1266 | 1.000 | .988 |
|  | .1299 | 1.000 | .982 |
|  | .1325 | 1.000 | .970 |
|  | .1342 | 1.000 | .967 |
|  | .1370 | 1.000 | .961 |
|  | .1409 | 1.000 | .958 |
|  | .1464 | 1.000 | .932 |
|  | .1519 | 1.000 | .926 |
|  | .1559 | 1.000 | .923 |
|  | .1589 | 1.000 | .917 |
|  | .1611 | 1.000 | .914 |
|  | .1644 | 1.000 | .908 |
|  | .1690 | 1.000 | .899 |
|  | .1732 | 1.000 | .893 |
|  | .1796 | 1.000 | .890 |
|  | .1859 | 1.000 | .887 |
|  | .1899 | 1.000 | .884 |
|  | .1934 | 1.000 | .881 |
|  | .1972 | 1.000 | .875 |
|  | .2000 | 1.000 | .872 |
|  | .2042 | 1.000 | .863 |
|  | .2153 | 1.000 | .860 |
|  | .2247 | 1.000 | .851 |
|  | .2327 | 1.000 | .848 |
|  | .2410 | 1.000 | .839 |
|  | .2442 | 1.000 | .836 |
|  | .2472 | 1.000 | .833 |
|  | .2532 | 1.000 | .682 |
|  | .2598 | 1.000 | .661 |
|  | .2667 | 1.000 | .622 |
|  | .2740 | 1.000 | .577 |
|  | .2817 | .987 | .491 |
|  | .2899 | .987 | .435 |
|  | .2971 | .987 | .411 |
|  | .3015 | .981 | .411 |
|  | .3054 | .981 | .405 |
|  | .3101 | .981 | .399 |
|  | .3163 | .981 | .390 |
|  | .3213 | .981 | .387 |
|  | .3231 | .981 | .372 |
|  | .3284 | .981 | .369 |
|  | .3381 | .968 | .190 |
|  | .3438 | .968 | .185 |
|  | .3489 | .968 | .182 |
|  | .3550 | .968 | .176 |
|  | .3591 | .968 | .164 |
|  | .3648 | .968 | .161 |
|  | .3694 | .968 | .155 |
|  | .3775 | .961 | .152 |
|  | .3868 | .955 | .140 |
|  | .3944 | .955 | .137 |
|  | .4000 | .955 | .134 |
|  | .4031 | .922 | .110 |
|  | .4083 | .922 | .107 |
|  | .4135 | .922 | .104 |
|  | .4189 | .922 | .101 |
|  | .4279 | .922 | .098 |
|  | .4447 | .916 | .095 |
|  | .4558 | .916 | .089 |
|  | .4629 | .916 | .086 |
|  | .4697 | .916 | .083 |
|  | .4734 | .916 | .080 |
|  | .4788 | .916 | .077 |
|  | .4821 | .916 | .074 |
|  | .4842 | .916 | .071 |
|  | .4929 | .916 | .068 |
|  | .5100 | .903 | .065 |
|  | .5303 | .903 | .063 |
|  | .5480 | .903 | .060 |
|  | .5616 | .903 | .057 |
|  | .5695 | .903 | .054 |
|  | .5857 | .903 | .051 |
|  | .6333 | .903 | .048 |
|  | .6762 | .903 | .042 |
|  | .6877 | .896 | .042 |
|  | .7020 | .890 | .042 |
|  | .7248 | .877 | .039 |
|  | .7380 | .877 | .036 |
|  | .7418 | .864 | .033 |
|  | .7464 | .857 | .033 |
|  | .7596 | .844 | .033 |
|  | .7752 | .844 | .030 |
|  | .7939 | .838 | .030 |
|  | .8086 | .831 | .030 |
|  | .8161 | .825 | .030 |
|  | .8250 | .818 | .030 |
|  | .8310 | .812 | .030 |
|  | .8426 | .799 | .027 |
|  | .8545 | .792 | .027 |
|  | .8596 | .786 | .027 |
|  | .8775 | .779 | .027 |
|  | .9010 | .740 | .027 |
|  | .9175 | .734 | .027 |
|  | .9307 | .714 | .027 |
|  | .9365 | .708 | .027 |
|  | .9449 | .701 | .027 |
|  | .9570 | .701 | .021 |
|  | .9623 | .688 | .021 |
|  | .9642 | .682 | .021 |
|  | .9666 | .675 | .021 |
|  | .9700 | .669 | .021 |
|  | .9861 | .662 | .021 |
|  | 1.0172 | .617 | .021 |
|  | 1.0358 | .604 | .021 |
|  | 1.0394 | .597 | .021 |
|  | 1.0426 | .584 | .021 |
|  | 1.0575 | .584 | .018 |
|  | 1.0913 | .539 | .018 |
|  | 1.1156 | .487 | .018 |
|  | 1.1314 | .474 | .018 |
|  | 1.1484 | .468 | .018 |
|  | 1.1569 | .429 | .018 |
|  | 1.1633 | .422 | .015 |
|  | 1.1833 | .409 | .015 |
|  | 1.2154 | .351 | .012 |
|  | 1.2404 | .344 | .012 |
|  | 1.2546 | .305 | .012 |
|  | 1.2778 | .299 | .012 |
|  | 1.3003 | .292 | .012 |
|  | 1.3060 | .286 | .012 |
|  | 1.3205 | .279 | .012 |
|  | 1.3397 | .240 | .012 |
|  | 1.3573 | .221 | .012 |
|  | 1.3787 | .214 | .012 |
|  | 1.3944 | .208 | .009 |
|  | 1.4083 | .201 | .009 |
|  | 1.4199 | .195 | .009 |
|  | 1.4258 | .188 | .009 |
|  | 1.4343 | .182 | .009 |
|  | 1.4450 | .169 | .006 |
|  | 1.4542 | .162 | .006 |
|  | 1.4699 | .149 | .006 |
|  | 1.4907 | .143 | .006 |
|  | 1.5119 | .130 | .006 |
|  | 1.5311 | .123 | .006 |
|  | 1.5518 | .117 | .006 |
|  | 1.5826 | .110 | .006 |
|  | 1.6214 | .091 | .006 |
|  | 1.6548 | .084 | .006 |
|  | 1.6852 | .078 | .006 |
|  | 1.7172 | .065 | .006 |
|  | 1.7404 | .058 | .006 |
|  | 1.7750 | .045 | .006 |
|  | 1.8524 | .032 | .003 |
|  | 1.9524 | .026 | .003 |
|  | 2.1250 | .013 | .003 |
|  | 2.3355 | .013 | .000 |
|  | 2.4605 | .006 | .000 |
|  | 3.5000 | .000 | .000 |
| The test result variable(s): Platelets, C-reactive protein, Lactate, Albumin, L/A ratio has at least one tie between the positive actual state group and the negative actual state group. | | | |
| a. The smallest cutoff value is the minimum observed test value minus 1, and the largest cutoff value is the maximum observed test value plus 1. All the other cutoff values are the averages of two consecutive ordered observed test values. | | | |

**Supplemental Table 2. Biomarker performance and cut-offs for mortality**

| **Coordinates of the Curve** | | | |
| --- | --- | --- | --- |
| Test Result Variable(s) | Positive if Greater Than or Equal To^a^ | Sensitivity | 1 - Specificity |
| Platelets | 79.00 | 1.000 | 1.000 |
|  | 82.50 | .984 | .997 |
|  | 86.50 | .978 | .997 |
|  | 94.00 | .967 | .997 |
|  | 104.00 | .923 | .993 |
|  | 109.00 | .923 | .990 |
|  | 111.00 | .885 | .990 |
|  | 112.50 | .880 | .990 |
|  | 116.50 | .880 | .987 |
|  | 122.00 | .852 | .987 |
|  | 127.00 | .852 | .984 |
|  | 133.00 | .847 | .984 |
|  | 136.50 | .842 | .984 |
|  | 138.50 | .842 | .980 |
|  | 141.50 | .831 | .974 |
|  | 143.50 | .831 | .971 |
|  | 144.50 | .831 | .967 |
|  | 145.50 | .825 | .954 |
|  | 146.50 | .820 | .954 |
|  | 147.50 | .820 | .951 |
|  | 149.00 | .814 | .948 |
|  | 150.50 | .803 | .928 |
|  | 152.00 | .798 | .925 |
|  | 153.50 | .787 | .922 |
|  | 154.50 | .770 | .915 |
|  | 155.50 | .760 | .906 |
|  | 156.50 | .754 | .902 |
|  | 157.50 | .743 | .902 |
|  | 158.50 | .743 | .899 |
|  | 159.50 | .743 | .896 |
|  | 160.50 | .738 | .896 |
|  | 161.50 | .738 | .893 |
|  | 163.50 | .732 | .889 |
|  | 166.00 | .732 | .886 |
|  | 168.50 | .732 | .883 |
|  | 171.00 | .732 | .870 |
|  | 172.50 | .727 | .866 |
|  | 174.50 | .727 | .863 |
|  | 181.00 | .727 | .853 |
|  | 186.50 | .727 | .847 |
|  | 188.50 | .727 | .840 |
|  | 191.50 | .721 | .840 |
|  | 193.50 | .721 | .837 |
|  | 195.50 | .721 | .824 |
|  | 198.50 | .716 | .824 |
|  | 200.50 | .661 | .779 |
|  | 201.50 | .661 | .772 |
|  | 203.00 | .661 | .769 |
|  | 207.00 | .661 | .762 |
|  | 211.00 | .634 | .743 |
|  | 215.00 | .634 | .739 |
|  | 219.00 | .634 | .733 |
|  | 220.50 | .634 | .726 |
|  | 222.00 | .623 | .707 |
|  | 224.00 | .617 | .707 |
|  | 225.50 | .617 | .704 |
|  | 226.50 | .617 | .697 |
|  | 228.50 | .612 | .697 |
|  | 230.50 | .596 | .691 |
|  | 232.00 | .574 | .678 |
|  | 233.50 | .574 | .668 |
|  | 234.50 | .563 | .642 |
|  | 235.50 | .557 | .632 |
|  | 236.50 | .557 | .629 |
|  | 237.50 | .557 | .619 |
|  | 239.00 | .552 | .616 |
|  | 240.50 | .492 | .560 |
|  | 241.50 | .486 | .544 |
|  | 242.50 | .475 | .534 |
|  | 243.50 | .432 | .518 |
|  | 244.50 | .426 | .518 |
|  | 245.50 | .339 | .440 |
|  | 247.50 | .333 | .436 |
|  | 249.50 | .328 | .436 |
|  | 253.00 | .295 | .423 |
|  | 256.50 | .295 | .420 |
|  | 261.50 | .295 | .414 |
|  | 269.00 | .290 | .414 |
|  | 273.00 | .284 | .414 |
|  | 274.50 | .279 | .414 |
|  | 275.50 | .273 | .414 |
|  | 276.50 | .273 | .410 |
|  | 278.50 | .273 | .404 |
|  | 280.50 | .273 | .401 |
|  | 281.50 | .273 | .397 |
|  | 282.50 | .273 | .394 |
|  | 284.00 | .273 | .391 |
|  | 285.50 | .273 | .388 |
|  | 287.00 | .273 | .384 |
|  | 288.50 | .262 | .384 |
|  | 289.50 | .257 | .384 |
|  | 292.00 | .257 | .371 |
|  | 295.00 | .251 | .371 |
|  | 298.00 | .251 | .368 |
|  | 304.50 | .224 | .313 |
|  | 309.50 | .219 | .313 |
|  | 311.00 | .208 | .306 |
|  | 315.50 | .208 | .303 |
|  | 320.00 | .208 | .300 |
|  | 323.00 | .197 | .283 |
|  | 325.50 | .197 | .280 |
|  | 327.00 | .197 | .277 |
|  | 328.50 | .191 | .270 |
|  | 330.00 | .191 | .267 |
|  | 331.50 | .191 | .264 |
|  | 333.00 | .191 | .261 |
|  | 334.50 | .191 | .257 |
|  | 337.00 | .191 | .254 |
|  | 339.50 | .191 | .251 |
|  | 340.50 | .169 | .215 |
|  | 341.50 | .164 | .215 |
|  | 342.50 | .158 | .208 |
|  | 343.50 | .148 | .199 |
|  | 344.50 | .148 | .192 |
|  | 345.50 | .098 | .147 |
|  | 347.50 | .098 | .134 |
|  | 349.50 | .093 | .130 |
|  | 351.00 | .087 | .107 |
|  | 352.50 | .087 | .104 |
|  | 353.50 | .087 | .101 |
|  | 354.50 | .087 | .098 |
|  | 357.50 | .087 | .094 |
|  | 360.50 | .082 | .094 |
|  | 362.50 | .082 | .091 |
|  | 365.00 | .082 | .088 |
|  | 368.00 | .077 | .088 |
|  | 371.00 | .077 | .085 |
|  | 375.00 | .077 | .081 |
|  | 384.00 | .077 | .078 |
|  | 392.00 | .077 | .075 |
|  | 397.00 | .071 | .075 |
|  | 402.50 | .060 | .072 |
|  | 406.50 | .060 | .068 |
|  | 409.50 | .055 | .068 |
|  | 417.50 | .049 | .068 |
|  | 427.50 | .049 | .065 |
|  | 432.00 | .049 | .062 |
|  | 434.00 | .049 | .059 |
|  | 437.50 | .049 | .055 |
|  | 443.50 | .044 | .052 |
|  | 448.50 | .044 | .042 |
|  | 458.50 | .038 | .039 |
|  | 476.50 | .038 | .036 |
|  | 489.50 | .033 | .036 |
|  | 494.50 | .027 | .036 |
|  | 498.00 | .027 | .033 |
|  | 504.50 | .022 | .033 |
|  | 517.50 | .022 | .029 |
|  | 532.00 | .022 | .026 |
|  | 563.50 | .016 | .026 |
|  | 594.50 | .016 | .023 |
|  | 602.00 | .016 | .020 |
|  | 608.50 | .016 | .016 |
|  | 623.00 | .016 | .013 |
|  | 637.00 | .016 | .010 |
|  | 653.50 | .011 | .010 |
|  | 669.50 | .011 | .007 |
|  | 703.00 | .000 | .007 |
|  | 745.50 | .000 | .003 |
|  | 759.00 | .000 | .000 |
| C-reactive protein | .5000 | 1.000 | 1.000 |
|  | .5500 | 1.000 | .997 |
|  | .6500 | 1.000 | .993 |
|  | .8500 | 1.000 | .990 |
|  | 1.2000 | 1.000 | .987 |
|  | 1.6500 | 1.000 | .984 |
|  | 1.9500 | 1.000 | .977 |
|  | 2.0500 | 1.000 | .971 |
|  | 2.2000 | 1.000 | .967 |
|  | 2.4000 | .995 | .967 |
|  | 2.5500 | .995 | .964 |
|  | 2.7000 | .989 | .945 |
|  | 2.9000 | .989 | .935 |
|  | 3.1500 | .989 | .919 |
|  | 3.3500 | .984 | .919 |
|  | 3.4500 | .978 | .919 |
|  | 3.6000 | .978 | .915 |
|  | 3.8000 | .978 | .912 |
|  | 3.9500 | .978 | .902 |
|  | 4.1000 | .973 | .879 |
|  | 4.2500 | .973 | .876 |
|  | 4.4000 | .967 | .873 |
|  | 4.7500 | .962 | .870 |
|  | 5.1500 | .956 | .821 |
|  | 5.3500 | .956 | .811 |
|  | 5.4500 | .956 | .808 |
|  | 5.6000 | .956 | .798 |
|  | 5.8000 | .951 | .792 |
|  | 5.9500 | .951 | .785 |
|  | 6.0500 | .940 | .779 |
|  | 6.3000 | .940 | .775 |
|  | 6.5500 | .934 | .772 |
|  | 6.8000 | .934 | .769 |
|  | 7.0500 | .934 | .762 |
|  | 7.3000 | .934 | .759 |
|  | 7.6500 | .934 | .756 |
|  | 7.9000 | .929 | .749 |
|  | 8.1500 | .929 | .726 |
|  | 8.3500 | .929 | .723 |
|  | 8.4500 | .923 | .717 |
|  | 8.5500 | .918 | .707 |
|  | 8.7500 | .913 | .704 |
|  | 8.9500 | .913 | .694 |
|  | 9.5000 | .907 | .678 |
|  | 10.5000 | .902 | .655 |
|  | 11.5000 | .902 | .645 |
|  | 12.5000 | .858 | .544 |
|  | 13.5000 | .836 | .515 |
|  | 14.5000 | .765 | .388 |
|  | 15.4500 | .678 | .329 |
|  | 15.9500 | .678 | .326 |
|  | 16.1000 | .672 | .296 |
|  | 16.2500 | .667 | .296 |
|  | 16.6500 | .656 | .296 |
|  | 17.5000 | .628 | .287 |
|  | 18.5000 | .601 | .274 |
|  | 19.1000 | .557 | .267 |
|  | 19.3500 | .557 | .264 |
|  | 19.7000 | .552 | .264 |
|  | 19.9500 | .552 | .261 |
|  | 20.2500 | .546 | .254 |
|  | 20.7500 | .541 | .254 |
|  | 21.1000 | .530 | .238 |
|  | 21.6000 | .530 | .235 |
|  | 22.1500 | .519 | .225 |
|  | 22.6500 | .514 | .221 |
|  | 23.4000 | .503 | .212 |
|  | 23.9000 | .492 | .212 |
|  | 24.5000 | .475 | .202 |
|  | 25.2500 | .459 | .192 |
|  | 25.5500 | .454 | .189 |
|  | 25.8000 | .454 | .182 |
|  | 26.5000 | .448 | .182 |
|  | 27.4500 | .443 | .176 |
|  | 27.9500 | .437 | .169 |
|  | 28.5000 | .426 | .163 |
|  | 29.5000 | .421 | .160 |
|  | 30.5000 | .421 | .156 |
|  | 31.4500 | .415 | .156 |
|  | 31.9500 | .415 | .153 |
|  | 32.0500 | .383 | .117 |
|  | 32.5500 | .372 | .114 |
|  | 33.1500 | .372 | .111 |
|  | 33.6500 | .366 | .111 |
|  | 34.5000 | .251 | .085 |
|  | 35.4000 | .219 | .085 |
|  | 35.9000 | .213 | .085 |
|  | 36.5000 | .208 | .085 |
|  | 37.1500 | .202 | .078 |
|  | 38.1500 | .202 | .075 |
|  | 39.5000 | .197 | .072 |
|  | 41.5000 | .191 | .072 |
|  | 44.0000 | .186 | .065 |
|  | 45.1000 | .142 | .039 |
|  | 45.6000 | .142 | .036 |
|  | 48.0000 | .137 | .036 |
|  | 51.0000 | .131 | .033 |
|  | 53.0000 | .126 | .033 |
|  | 54.5000 | .120 | .033 |
|  | 55.5000 | .109 | .026 |
|  | 58.0000 | .098 | .026 |
|  | 65.0000 | .098 | .023 |
|  | 72.5000 | .087 | .023 |
|  | 75.5000 | .082 | .023 |
|  | 77.5000 | .077 | .023 |
|  | 79.5000 | .071 | .023 |
|  | 85.0000 | .060 | .023 |
|  | 120.0000 | .055 | .023 |
|  | 152.0000 | .049 | .023 |
|  | 177.0000 | .044 | .023 |
|  | 216.0000 | .038 | .023 |
|  | 260.0000 | .038 | .020 |
|  | 294.0000 | .038 | .016 |
|  | 320.0000 | .027 | .010 |
|  | 345.0000 | .027 | .007 |
|  | 377.5000 | .022 | .007 |
|  | 407.0000 | .016 | .007 |
|  | 429.5000 | .016 | .003 |
|  | 525.5000 | .011 | .003 |
|  | 602.0000 | .005 | .003 |
|  | 608.5000 | .005 | .000 |
|  | 615.0000 | .000 | .000 |
| Lactate | .4000 | 1.000 | 1.000 |
|  | .4500 | 1.000 | .987 |
|  | .5500 | .984 | .915 |
|  | .6500 | .978 | .879 |
|  | .7500 | .978 | .863 |
|  | .9000 | .967 | .857 |
|  | 1.0500 | .814 | .127 |
|  | 1.1500 | .814 | .121 |
|  | 1.2500 | .809 | .101 |
|  | 1.3500 | .798 | .091 |
|  | 1.4500 | .798 | .075 |
|  | 1.5500 | .798 | .059 |
|  | 1.6500 | .798 | .046 |
|  | 1.8500 | .798 | .042 |
|  | 2.0500 | .760 | .016 |
|  | 2.2000 | .760 | .013 |
|  | 2.3500 | .749 | .013 |
|  | 2.4500 | .727 | .013 |
|  | 2.5500 | .579 | .010 |
|  | 2.6500 | .563 | .010 |
|  | 2.7500 | .557 | .010 |
|  | 2.8500 | .525 | .010 |
|  | 2.9500 | .497 | .010 |
|  | 3.1000 | .246 | .007 |
|  | 3.3000 | .230 | .007 |
|  | 3.4500 | .213 | .007 |
|  | 3.5500 | .153 | .003 |
|  | 3.6500 | .115 | .000 |
|  | 3.8500 | .109 | .000 |
|  | 4.2500 | .044 | .000 |
|  | 4.5500 | .027 | .000 |
|  | 4.8000 | .005 | .000 |
|  | 6.0000 | .000 | .000 |
| Albumin | .5000 | 1.000 | 1.000 |
|  | 1.6500 | .995 | 1.000 |
|  | 1.8500 | .978 | 1.000 |
|  | 1.9500 | .967 | 1.000 |
|  | 2.0500 | .902 | .997 |
|  | 2.2000 | .880 | .993 |
|  | 2.3500 | .858 | .990 |
|  | 2.4500 | .792 | .987 |
|  | 2.5500 | .607 | .948 |
|  | 2.6500 | .492 | .938 |
|  | 2.7500 | .344 | .935 |
|  | 2.8500 | .235 | .915 |
|  | 2.9500 | .208 | .909 |
|  | 3.0500 | .137 | .717 |
|  | 3.1500 | .115 | .704 |
|  | 3.2500 | .098 | .681 |
|  | 3.3500 | .093 | .668 |
|  | 3.4500 | .087 | .629 |
|  | 3.5500 | .055 | .515 |
|  | 3.6500 | .044 | .384 |
|  | 3.7500 | .027 | .322 |
|  | 3.8500 | .022 | .251 |
|  | 3.9500 | .022 | .208 |
|  | 4.0500 | .005 | .026 |
|  | 4.1500 | .000 | .026 |
|  | 4.3000 | .000 | .016 |
|  | 4.4500 | .000 | .013 |
|  | 5.5000 | .000 | .000 |
| L/A ratio | .0900 | 1.000 | 1.000 |
|  | .1056 | 1.000 | .997 |
|  | .1181 | 1.000 | .993 |
|  | .1266 | 1.000 | .987 |
|  | .1299 | 1.000 | .980 |
|  | .1325 | 1.000 | .967 |
|  | .1342 | 1.000 | .964 |
|  | .1370 | 1.000 | .958 |
|  | .1409 | 1.000 | .954 |
|  | .1464 | 1.000 | .925 |
|  | .1519 | 1.000 | .919 |
|  | .1559 | 1.000 | .915 |
|  | .1589 | 1.000 | .909 |
|  | .1611 | 1.000 | .906 |
|  | .1644 | .995 | .902 |
|  | .1690 | .989 | .896 |
|  | .1732 | .989 | .889 |
|  | .1796 | .989 | .886 |
|  | .1859 | .989 | .883 |
|  | .1899 | .989 | .879 |
|  | .1934 | .984 | .879 |
|  | .1972 | .984 | .873 |
|  | .2000 | .984 | .870 |
|  | .2042 | .978 | .863 |
|  | .2153 | .978 | .860 |
|  | .2247 | .978 | .850 |
|  | .2327 | .978 | .847 |
|  | .2410 | .978 | .837 |
|  | .2442 | .973 | .837 |
|  | .2472 | .973 | .834 |
|  | .2532 | .967 | .671 |
|  | .2598 | .967 | .648 |
|  | .2667 | .962 | .609 |
|  | .2740 | .956 | .564 |
|  | .2817 | .945 | .469 |
|  | .2899 | .934 | .414 |
|  | .2971 | .929 | .391 |
|  | .3015 | .923 | .391 |
|  | .3054 | .923 | .384 |
|  | .3101 | .923 | .378 |
|  | .3163 | .923 | .368 |
|  | .3213 | .918 | .368 |
|  | .3231 | .913 | .355 |
|  | .3284 | .913 | .352 |
|  | .3381 | .880 | .169 |
|  | .3438 | .880 | .163 |
|  | .3489 | .880 | .160 |
|  | .3550 | .880 | .153 |
|  | .3591 | .880 | .140 |
|  | .3648 | .880 | .137 |
|  | .3694 | .880 | .130 |
|  | .3775 | .869 | .130 |
|  | .3868 | .852 | .124 |
|  | .3944 | .852 | .121 |
|  | .4000 | .852 | .117 |
|  | .4031 | .825 | .091 |
|  | .4083 | .825 | .088 |
|  | .4135 | .825 | .085 |
|  | .4189 | .825 | .081 |
|  | .4279 | .825 | .078 |
|  | .4447 | .820 | .075 |
|  | .4558 | .820 | .068 |
|  | .4629 | .820 | .065 |
|  | .4697 | .820 | .062 |
|  | .4734 | .820 | .059 |
|  | .4788 | .820 | .055 |
|  | .4821 | .814 | .055 |
|  | .4842 | .814 | .052 |
|  | .4929 | .814 | .049 |
|  | .5100 | .798 | .049 |
|  | .5303 | .798 | .046 |
|  | .5480 | .798 | .042 |
|  | .5616 | .798 | .039 |
|  | .5695 | .798 | .036 |
|  | .5857 | .798 | .033 |
|  | .6333 | .798 | .029 |
|  | .6762 | .798 | .023 |
|  | .6877 | .792 | .023 |
|  | .7020 | .787 | .023 |
|  | .7248 | .776 | .020 |
|  | .7380 | .776 | .016 |
|  | .7418 | .765 | .013 |
|  | .7464 | .760 | .013 |
|  | .7596 | .749 | .013 |
|  | .7752 | .749 | .010 |
|  | .7939 | .743 | .010 |
|  | .8086 | .738 | .010 |
|  | .8161 | .732 | .010 |
|  | .8250 | .727 | .010 |
|  | .8310 | .721 | .010 |
|  | .8426 | .705 | .010 |
|  | .8545 | .699 | .010 |
|  | .8596 | .694 | .010 |
|  | .8775 | .689 | .010 |
|  | .9010 | .656 | .010 |
|  | .9175 | .650 | .010 |
|  | .9307 | .634 | .010 |
|  | .9365 | .628 | .010 |
|  | .9449 | .623 | .010 |
|  | .9570 | .612 | .010 |
|  | .9623 | .601 | .010 |
|  | .9642 | .596 | .010 |
|  | .9666 | .590 | .010 |
|  | .9700 | .585 | .010 |
|  | .9861 | .585 | .007 |
|  | 1.0172 | .546 | .007 |
|  | 1.0358 | .536 | .007 |
|  | 1.0394 | .530 | .007 |
|  | 1.0426 | .519 | .007 |
|  | 1.0575 | .514 | .007 |
|  | 1.0913 | .481 | .003 |
|  | 1.1156 | .437 | .003 |
|  | 1.1314 | .426 | .003 |
|  | 1.1484 | .421 | .003 |
|  | 1.1569 | .388 | .003 |
|  | 1.1633 | .377 | .003 |
|  | 1.1833 | .366 | .003 |
|  | 1.2154 | .311 | .003 |
|  | 1.2404 | .306 | .003 |
|  | 1.2546 | .273 | .003 |
|  | 1.2778 | .268 | .003 |
|  | 1.3003 | .262 | .003 |
|  | 1.3060 | .257 | .003 |
|  | 1.3205 | .251 | .003 |
|  | 1.3397 | .219 | .003 |
|  | 1.3573 | .202 | .003 |
|  | 1.3787 | .197 | .003 |
|  | 1.3944 | .186 | .003 |
|  | 1.4083 | .180 | .003 |
|  | 1.4199 | .175 | .003 |
|  | 1.4258 | .169 | .003 |
|  | 1.4343 | .164 | .003 |
|  | 1.4450 | .148 | .003 |
|  | 1.4542 | .142 | .003 |
|  | 1.4699 | .131 | .003 |
|  | 1.4907 | .126 | .003 |
|  | 1.5119 | .115 | .003 |
|  | 1.5311 | .109 | .003 |
|  | 1.5518 | .104 | .003 |
|  | 1.5826 | .098 | .003 |
|  | 1.6214 | .082 | .003 |
|  | 1.6548 | .077 | .003 |
|  | 1.6852 | .071 | .003 |
|  | 1.7172 | .060 | .003 |
|  | 1.7404 | .055 | .003 |
|  | 1.7750 | .044 | .003 |
|  | 1.8524 | .033 | .000 |
|  | 1.9524 | .027 | .000 |
|  | 2.1250 | .016 | .000 |
|  | 2.3355 | .011 | .000 |
|  | 2.4605 | .005 | .000 |
|  | 3.5000 | .000 | .000 |
| The test result variable(s): Platelets, C-reactive protein, Lactate, Albumin, L/A ratio has at least one tie between the positive actual state group and the negative actual state group. | | | |
| a. The smallest cutoff value is the minimum observed test value minus 1, and the largest cutoff value is the maximum observed test value plus 1. All the other cutoff values are the averages of two consecutive ordered observed test values. | | | |
